# Use of *Salmonella* Bacteria in Cancer Therapy: Direct, Drug Delivery and Combination Approaches

**DOI:** 10.3389/fonc.2021.624759

**Published:** 2021-03-02

**Authors:** Fereshteh Badie, Maryam Ghandali, Seyed Alireza Tabatabaei, Mahmood Safari, Ahmad Khorshidi, Mohammad Shayestehpour, Maryam Mahjoubin-Tehran, Korosh Morshedi, Amin Jalili, Vida Tajiknia, Michael R. Hamblin, Hamed Mirzaei

**Affiliations:** ^1^Department of Microbiology, Faculty of Medicine, Kashan University of Medical Sciences, Kashan, Iran; ^2^School of Medicine, Iran University of Medical Sciences, Tehran, Iran; ^3^Department of Internal Medicine, School of Medicine, Shahid Beheshti University of Medical Science, Tehran, Iran; ^4^Student Research Committee, Mashhad University of Medical Sciences, Mashhad, Iran; ^5^Department of Medical Biotechnology, Faculty of Medicine, Mashhad University of Medical Sciences, Mashhad, Iran; ^6^School of Medicine, Kashan University of Medical Sciences, Kashan, Iran; ^7^Department of Surgery, School of Medicine, Iran University of Medical Sciences, Tehran, Iran; ^8^Laser Research Centre, Faculty of Health Science, University of Johannesburg, Doornfontein, South Africa; ^9^Research Center for Biochemistry and Nutrition in Metabolic Diseases, Institute for Basic Sciences, Kashan University of Medical Sciences, Kashan, Iran

**Keywords:** bacteria-mediated cancer therapy, *Salmonella* species, cancer, drug delivery, therapy

## Abstract

Over the years, conventional cancer treatments, such as chemotherapy with only a limited specificity for tumors, have undergone significant improvement. Moreover, newer therapies such as immunotherapy have undergone a revolution to stimulate the innate as well as adaptive immune responses against the tumor. However, it has been found that tumors can be selectively colonized by certain bacteria, where they can proliferate, and exert direct oncolytic effects as well as stimulating the immune system. Bacterial-mediated cancer therapy (BMCT) is now one example of a hot topic in the antitumor field. *Salmonella typhimurium* is a Gram-negative species that generally causes self-limiting gastroenteritis in humans. This species has been designed and engineered in order to be used in cancer-targeted therapeutics. *S. typhimurium* can be used in combination with other treatments such as chemotherapy or radiotherapy for synergistic modification of the tumor microenvironment. Considerable benefits have been shown by using engineered attenuated strains for the diagnosis and treatment of tumors. Some of these treatment approaches have received FDA approval for early-phase clinical trials. This review summarizes the use of *Salmonella* bacteria for cancer therapy, which could pave the way towards routine clinical application. The benefits of this therapy include an automatic self-targeting ability, and the possibility of genetic manipulation to produce newly engineered attenuated strains. Nevertheless, *Salmonella-*mediated anticancer therapy has not yet been clinically established, and requires more research before its use in cancer treatment.

## Introduction

Recently, bacteria-mediated cancer therapy (BMCT) has attracted attention, in which different attenuated bacteria have been created using genetic engineering, and are capable of targeting tumors and exerting various antitumor effects (1). For more than a century bacteria have been investigated as a therapeutic approach to cancer (1). The bone surgeon William B. Coley was the first to report that injection of a preparation of heat-killed *Streptococcus pyogenes* into patients with inoperable bone and soft-tissue sarcomas could produce tumor regression (2). The treatment sometimes led to successful shrinkage of the tumors and extended the survival of the patients (3). Nevertheless, over the years, the lack of understanding of the basic mechanisms, and inadequate procedures and methods for diagnosis and quantification of the immune responses, resulted in a disappointing level of acceptance by the medical community. Nowadays, due to advances in medical technology (particularly in genetic engineering), BMCT has attracted more attention, and a range of different species of bacteria, including *Streptococcus* (4), *Bifidobacterium* (5), *Clostridium* (6), and *Salmonella* species (7, 8), have been investigated.

*Salmonella enterica serovar Typhimurium* (*S. typhimurium*) is capable of growth in both aerobic and anaerobic environments, and is therefore able to target and colonize both non-hypoxic and hypoxic tumors, along with metastatic tumor deposits which are accessible through the circulatory system. *S. typhimurium* can serve as a novel antitumor therapy to fill an existing gap, because radiotherapy and chemotherapy are known to be less effective in necrotic and hypoxic regions of tumors. Preferential accumulation and colonization of *S. typhimurium* takes place in tumors, leading to tumor-to-normal tissue ratios being measured, which can exceed 1,000–10,000 to 1. *Salmonella* species have shown the highest efficiency as an antitumor bacterial strain in the empirical models of cancer that have been tested up to now (9). *Salmonella*-based immunotherapy can be thought of as possessing “Trojan horse” properties, and can also be used in tumors, which have developed resistance to conventional treatments (1, 3, 10). Why do *S. typhimurium* bacterial strains possess tumor-targeting as well as tumor-destroying properties? The present review discusses these questions and provides some possible answers.

Moreover, *S. typhimurium*-based therapy can be effectively combined with radiotherapy (11) or chemotherapy (12). *Salmonella*-mediated antitumor therapy could be a new and effective approach in the treatment of different cancers.

## Treatment of Cancer Using Bacteria

One of the challenges in conventional cancer therapy is that anticancer drugs generally show restricted penetration into tumor tissue. Not only chemotherapy, but also other biological treatments such as monoclonal antibodies and cytokines, are limited by the passive transport of the molecules into the tumor. This consideration not only limits their effectiveness, but if the doses are increased to compensate, it leads to higher risks of toxicity (13).

Recently, some new therapeutic approaches have been developed based on microorganisms. Talimogene laherparepvec (T-VEC; Imlygic™) was approved by the US FDA for the treatment of advanced melanoma. T-VEC is a genetically modified herpes simplex virus, type 1. T-VEC was attenuated by the deletion of the herpes neurovirulence viral genes and enhanced for immunogenicity by the deletion of the viral ICP47 gene (14).

However, bacterial therapy relies on the intrinsic properties of living organisms to penetrate and accumulate in tumors, which are not available with traditional methods, making bacterial therapy promising for the future (13).

In the 19^th^ century, bacterial therapy was known for some adverse effects related to over-stimulation of the immune system, including fever, septic shock, and even death (15, 16). Advances in genetic engineering have resulted in the ability to use genetically modified bacteria, thus reducing their off-target toxicity for the treatment of cancer. The ability to manipulate the bacterial genome has made them a favorable option compared to other microorganisms (17).

BMCT has attracted much attention because of its considerable benefits. For instance, bacteria have the capability to penetrate into cancer tissue, showing selectivity towards tumor cells based on specific chemical signals that are enriched within the tumor microenvironment (18, 19). Moreover, bacteria can function as vectors for transport of therapeutic molecules and as drug delivery vehicles. Rationally designed bacteria can be controlled from the outside by administration of agents that only affect the microorganisms themselves and not the host tissue (20, 21). Interestingly, the concept of “artificial medical bacteria” could also contribute to the process of diagnosis (detection of molecules or tumor markers associated with specific diseases), and to the preparation of smart therapeutics (by responding to chemical stimuli by releasing therapeutic agents) (22). The design and construction of “living biological robots” may be possible with the use of synthetic biology to modify bacteria. A wide variety of agents can be integrated into laboratory-designed bacteria, such as genes, proteins, and drugs which could originally come from different organisms, but could now display satisfactory safety and efficacy against human cancer (23). Due to the tumor-tropic properties of bacteria they can be administered systemically. Accordingly, lower concentrations are required, and complicated procedures for purification or formulation may not be needed (22). Nevertheless, BMCT does have some limitations, such as biosafety, genetic instability, and the possible interaction of the bacteria with therapeutic drugs. The payload capacity of bacteria may be limited, leading to oncolytic viruses being preferred as a cancer therapy. Nevertheless, oncolytic viruses also have their own limitations, such as easy recognition by the immune system, and questions about an adequate level of biosafety (24, 25).

## *Salmonella* Bacteria and Treatment of Cancer

**Figure 1:**
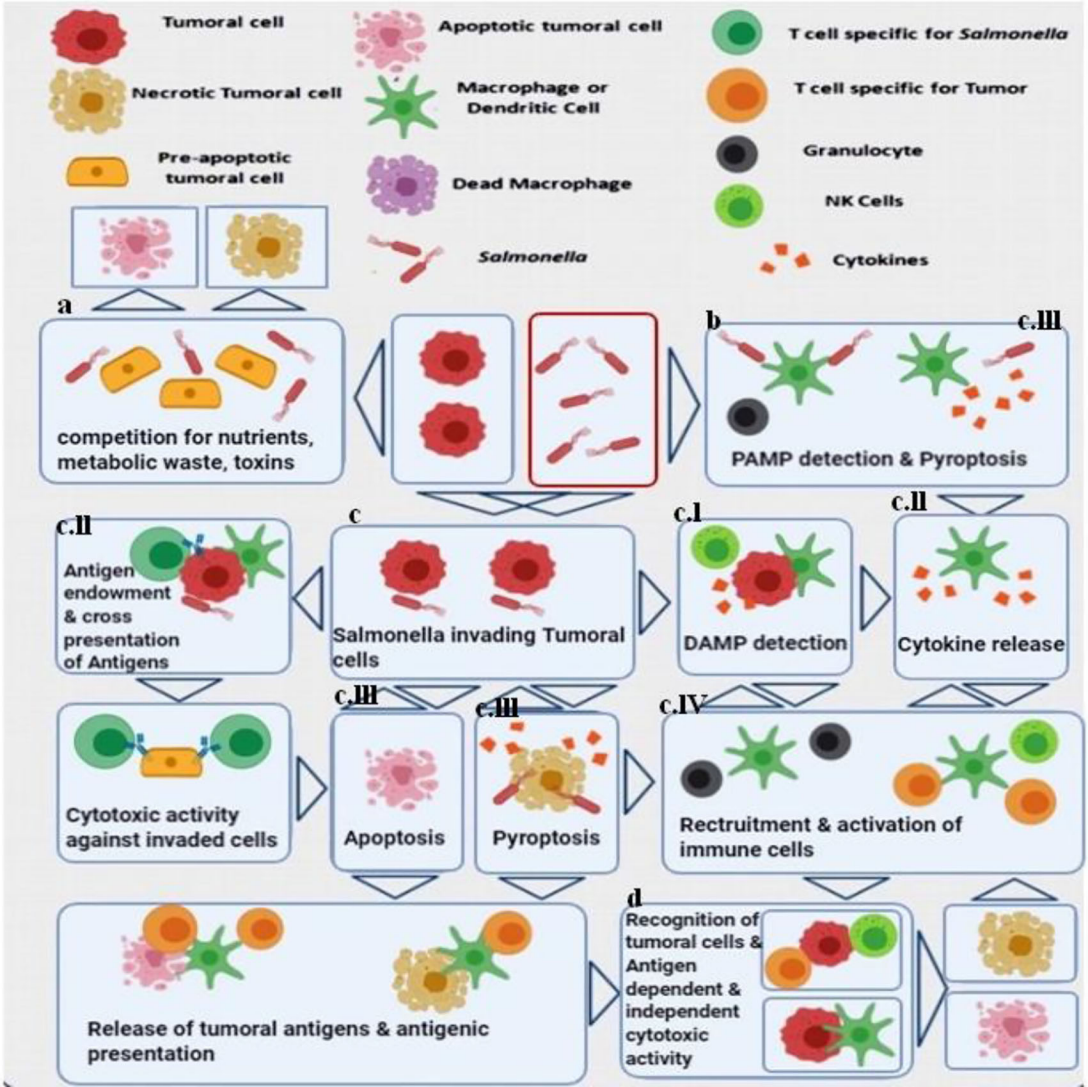
Major antitumor mechanisms stimulated by *Salmonella* (*S. typhimurium*). Both bacteria-mediated direct cytotoxicity and immune system-mediated indirect tumor cell death have been proposed. **(A)** In the tumor microenvironment, bacterial infection inhibits the growth of the tumor and results in considerable cell death. **(B)** The recognition of pathogen-related molecular patterns (PAMP) on the bacterial cells by receptors expressed on immune cells, triggers the release of cytokines and the recruitment of leukocytes that can initiate anti-tumor immune responses (29–31). **(C)** *S. typhimurium* uses a Type III secretion system which leads to the release of different factors into cancer cells, and the bacteria can also be internalized and undergo intra-cellular replication (32, 33). **(C.I)** Cell stress responses are induced by invasive *Salmonella* cells mediated *via* danger-associated molecular patterns (DAMP) that also act as signals to the immune system. **(C.II)** At the same time, this process together with the released cytokines leads to the presentation of bacterial antigens as well as cancer specific antigens, to the adaptive immune system leading to the production of cancer-antigen specific T-cells that can recognize and kill the cancer cells as well as the bacteria (34). An immunological synapse occurs between the bacteria, the cancer cells and the host dendritic cells leading to cross-presentation of tumor antigens, resulting in an antigen-specific anti-cancer immune response (35, 36). **(C.III)** *Salmonella* bacteria have the potential to directly cause cancer cell death through induction of apoptosis or pyroptosis. Pyroptosis is a form of programmed inflammatory cell death, characterized by activation of caspase 1 and the inflammasomes, and secretion of IL-1β as well as IL-18. It is also characterized by cell rounding and detachment, reorganization of the cytoskeleton, deformation of the nucleus, together with rupture of the cell membrane and release of additional inflammatory signals (37–39). The description of pyroptosis first took place in macrophages that died rapidly under certain conditions, which is particularly important in cancer immunotherapy, since tumor-associated macrophages (TAM) are considered to possess immune-suppressive properties. Reduction of TAMs can be considered as another aspect of the *S. typhimurium* anti-tumor activity. The dead cancer cells can liberate tumor antigens, while the surrounding cancer cells are infected by the released bacteria. **(C.IV)** During pyroptosis, immune cell recruitment and activation can be triggered by the pro-inflammatory cytokines IL-1β and IL-18 (37, 38, 40). **(D)** The convergence of several different mechanisms enables recognition of tumor antigens, leading to stimulation of both antigen-dependent and antigen-independent cell killing. The proteasomal degradation *of S. typhimurium* proteins within the cancer cell cytosol, leads to production of bacterial peptides that can be presented to cytotoxic lymphocytes through MHC I (34, 41). This figure is adapted from (42).

Research has been performed on different mutant strains of *S. typhimurium* that have been investigated for anti-cancer applications. Laboratory strains of *S. typhimurium* have been modified to reduce their toxicity to normal organs, while increasing their specific targeting properties for tumors along with enhancing their antitumor activity (26–28). **Figure 1** displays a complicated series of processes that may be involved in the anti-tumor effectiveness of *Salmonella* species.

## *Salmonella* Bacteria as Delivery Systems for Anti-Tumor Agents

Ovarian cancer is a gynecological tumor with considerable morbidity and mortality (43, 44). Chemotherapy and surgery are the main treatments for ovarian cancer, but because of multidrug resistance (MDR) these approaches are not completely successful. Tumors develop MDR as a defense mechanism against cytotoxic drugs, which causes chemotherapy failure in many patients. The most important mechanism of MDR is the production of the plasma membrane glycoprotein (170 KDaP-gp) that binds to ATP and can then pump cytotoxic drugs out of the cells, thus reducing the concentration to a sublethal level. P-gp is a highly conserved membrane glycoprotein encoded by the *MDR1* gene. In one study, Deng et al., assessed the effects of attenuated *Salmonella typhi* acting as a tumor-targeted vector to deliver small interfering RNA (siRNA) against the multidrug-resistance gene (MDR1) (45). This was tested in the cisplatin-resistant ovarian cancer cell line SKOV-3/DDP, to increase cisplatin sensitivity. For this purpose a MDR1 siRNA expression plasmid was constructed that contained short hairpin RNA (shRNA) against the MDR1 gene, which was loaded into cells of the *S. typhi* strain (SL7207). MDR1 gene expression was measured with real-time PCR and protein expression by Western blotting. BALB/c nude mice were subcutaneously injected with SKOV-3/DDP cells and orally inoculated with *Salmonella* containing MDR1 siRNA plasmid, and then intraperitoneally injected with cisplatin. It was found that the high level of MDR1 in SKOV-3/DDP cells was reduced by the bacteria, and the cisplatin resistance was reversed. The tumor growth in mice was slower when treated with DDP. Attenuated *Salmonella* bacteria could be used for delivery of RNA interference as a component of cancer treatment (45).

In one study, tumor-bearing nude mice that were orally administered recombinant *Salmonella* displayed a slower tumor growth and became more sensitive to DDP. Deng et al. established a cisplatin (DDP)-resistant ovarian cancer cell line SKOV-3/DDP by treatment with gradually increasing concentrations of cisplatin. They found that compared with the parental cell line, the DDP-resistant SKOV-3/DDP cells expressed a much higher level of MDR1. The expression of MDR1 in SKOV-3/DDP cells infected with the Salmonella strain bearing MDR1 siRNA plasmid *in vitro* was found to be down-regulated and the DDP tolerance of these cells was reversed (45).

Epidermal growth factor receptor (EGFR) is often over-expressed in tumor cells (46). Transforming growth factor alpha (TGFα) is a naturally occurring ligand for the EGFR. PE38 is a recombinant immunotoxin prepared as a conjugate between TGFα and a laboratory-engineered *Pseudomonas* exotoxin A. The PE38 immunotoxin is under investigation for EGFR-positive cancers, for example brain tumors (47, 48). Pseudomonas exotoxin A suppresses protein production in mammalian cells (49). PE38 binds to EGFR-expressing cancer cells due to the TGFα fragment present in the recombinant fusion protein. Studies have shown that PE38 has a toxic effect on EGFR-expressing tumor cells, both *in vitro* as well as against tumors in a mouse model (50, 51). However, systemic injection of TGFα-PE38 has shown dose-dependent hepatotoxicity (51). In this study, the ΔppGpp *Salmonella* mutant that expressed recombinant TGFα-PE38 was investigated. The ΔppGpp *Salmonella* mutant neither attacks nor proliferates within mammalian cells (52, 53), but exerts its anti-tumoral effects by inducing the expression of pro-inflammatory cytokines from macrophages and neutrophils, such as IL-1β and TNFα (54). In this study, a plasmid was constructed with DNA encoding TGFα-PE38 and inserted into *Salmonella* cells. The mouse tumor experiment employed colon or breast tumors with high levels of EGFR expression. They used an inducible system based on the PBAD promoter from *E. coli* (induced by intraperitoneal injection of L-arabinose) (55). In order to export the TGFα-PE38 recombinant protein from the *Salmonella* cells, an engineered phage lysis system (56) or a bacterial membrane transport signal fused to the protein were investigated. Both approaches appeared to be equally effective. They found that TGFα-PE38 produced by the bacteria slowed tumor growth compared to non-engineered *Salmonella* alone (56). Treatment of the tumor cells with TGFα-PE38 increased the expression of EGFR in the cells and induced tumor cell apoptosis. This research showed that the effectiveness of immunotoxins for tumor treatment could be significantly improved if they were delivered using bacteria (57).

Solid tumors are often highly hypoxic in comparison to healthy tissue, and are therefore readily colonized by attenuated *S. typhimurium*. Anti-tumor effects can be obtained if the bacteria express cytotoxic genes or by invasive bacterial infection leading to apoptosis or necrosis of cancer cells (58, 59). However the antitumor effects of *S. typhimurium* in human tumors have been relatively weak, compared to those seen in tumors in animal models (60, 61). This disparity is likely due to the considerable complexity of human immune system, and possibly to over-attenuation of *S. typhimurium. Salmonella* can also induce apoptosis or necrosis of endothelial cells within the tumor microvessels, as well as a decrease in the level of VEGF and the number of CD31 positive cells. This anti-angiogenesis effect can further increase the hypoxia within the tumor (59, 62). Therefore colonization of the tumor by *Salmonella* could create a positive feedback loop leading to the creation of a more complete anaerobic environment within the tumor. Research has shown that the use of *Salmonella* in combination with an angiogenesis inhibitor showed better performance in a mouse tumor model of pancreatic cancer patient-derived orthotopic xenografts (PDOX) (63).

Traditional Chinese medicine has used the medicinal herb called *Tripterygium wilfordii* Hook F for centuries to treat inflammatory and autoimmune diseases (64). This herb contains a diterpenoid triepoxide called Triptolide, which has anti-inflammatory and anti-angiogenesis properties. Triptolide has been investigated for suppressing the growth and metastasis of solid tumors such as melanoma (65–67). Moreover, combinations of Triptolide as an anti-angiogenic drug (68) with chemotherapy drugs, such as cisplatin have shown synergistic benefits (69). *S. typhimurium* VNP20009 has been tested in experimental models (60, 61), but did not show convincing therapeutic effects (60, 68). In one study, Chen et al. attempted to overcome the poor tumor colonization of attenuated *Salmonella* (70). They combined the VNP20009 *Salmonella* strain with administration of tryptolide against murine melanoma tumors. It was found that tryptolide combined with VNP20009 was superior to VNP20009 alone in reducing the number of neutrophils in the melanoma, and therefore improved the colonization of *Salmonella* and produced extensive necrosis. The combination therapy reduced VEGF expression and inhibited angiogenesis and melanoma growth. They concluded that combination therapy with triptolide enhanced the antitumor effects of VNP20009 by inhibiting angiogenesis and stimulating the host immune response (70).

5-Fluorocytosine (5-FC) is a non-toxic cytosine analog that can be converted into 5-fluorouracil (5-FU) by the action of the enzyme cytosine deaminase. The conversion of 5-FU into 5-F-dUMP inside the cell blocks thymidylate synthase activity. 5-FUTP and 5-FdUTP can be produced by the insertion of enzymes into RNA and DNA (71) thus leading to cell death (72, 73). In one study, Mesa-Pereir et al. investigated the cytotoxic activity of a *Salmonella* strain equipped with a salicylate-inducible expression apparatus, which could modulate the expression of cytosine deaminase (74). Firstly, they introduced a binding site for the T7 phage gene 10 ribosome sequence and replaced the original GUG start codon with AUG to allow the inclusion of an *E. coli* coda gene under the Pm promoter. A 5-FU resistant *Salmonella* strain was created to increase the production of cytosine deaminase within the bacteria. A purD mutation in the producer strain was further developed to control the intracellular proliferation in the presence of adenine as well as to prevent intracellular death of *Salmonella*. This approach allowed the use of cytosine deaminase produced by *Salmonella* strains to kill tumor cells in the presence of 5-FU (74).

In cancer cells, as well as blood vessels that express the αvβ3 integrin during angiogenesis, the RGD peptide can specifically bind to the αvβ3 integrin. Bacteria can be engineered to express the peptide RGD-4C associated with the outer membrane protein A (OmpA) on the bacterial cell surface to increase the efficiency of tumor targeting (75). In this study, the tumor tropism of *S. typhimurium* containing a RGD peptide sequence (ACDCRGDCFCG) in the external region of OmpA was investigated. *Salmonella* with the RGD sequence strongly bound to αvβ3 expressing tumor cells, whereas its binding to cells without αvβ3 was weak. *In vivo* studies showed that the use of RGD-expressing *Salmonella* could produce regression in αvβ3-over-expressing cancer xenografts, human breast cancer (MDA-MB-231) and human melanoma (MDA-MB-435), and prolong survival in mice. Therefore, the expression of RGD peptides on the surface of *Salmonella* could improve tumor targeting (75).

An enzyme called asparaginase (L-ASNase), derived from *E. coli*, has been used to treat acute lymphocytic leukemia (76). The function of L-ASNase is to catalyze the conversion of asparagine to aspartic acid, and to some extent glutamine to glutamate (77). Both reactions may be helpful for the treatment of cancer (78). When the asparagine concentration is lowered, uncharged tRNA activates the serine/threonine kinase GCN2 (79). eIF2á, is then phosphorylated by GCN2, and in turn inhibits the guanine nucleotide exchange factor eIF2B. This can block eIF2 recycling and inhibit global protein synthesis (80). If the resynthesis of asparagine is insufficient to keep the tRNA fully charged, the reduced rate of total protein synthesis triggers cell death due to apoptosis (81).

Kim et al. (82) devised a treatment for acute lymphoblastic leukemia using L-ASNase expressed in *Salmonella* bacteria. The araBAD *E. coli* inducible promoter was used to design *Salmonella* cells capable of delivering L-ASNase to tumor tissues (82). **Table 1** lists various anti-tumor agents that have been targeted or delivered by *Salmonella* strains.

**Table 1:** Anti-tumor agents that have been delivered or targeted by *Salmonella* strains.

Salmonella species	Type of cancer	Anti-tumor agent	Anti-cancer effect	Model	Type of cell line	Ref
*Salmonella typhi SL7207*	Ovarian cancer	RNA interference	Reduction of MDR1	SKOV-3/DDP tumor-bearing mice	SKOV-3/DDP	(83)
*Salmonella typhimurium*		shIDO	Apoptosis of tumor	Mice bearing B16F10 tumors		(84)
*Salmonella typhimurium VNP20009*	Lung cancer	Sox2shRNA	Anti-angiogenesis	Mice bearing A549 tumors		(85)
*Salmonella typhimurium*	Tumor	PLK1		Nude mice bearing human MDA-MB-231 xenografts		(86)
*Salmonella typhimurium*	Prostate tumor	Stat3-specific	Treatment of primary and metastatic cancer	C57BL6 mice bearing an implanted prostate tumor		(87)
*Salmonella typhimurium*	Hepatocellular carcinoma	Stat3-shRNA		Ectopic transplanted model of C57BL6 mice		(88)
*Salmonella typhimurium SC36*	Melanomas or pulmonary tumor	PNR	Apoptosis	Mice bearing melanomas or pulmonary tumors		(89)
*Salmonella typhimurium SC36*	Mammary carcinoma	PNR	Suicide gene/prodrug therapy	Mice bearing mammary carcinoma		(90)
*Salmonella typhimurium VNP20009*	Melanoma	PNR	Delayed tumor growth; increased CD8(+) T-cell infiltration	Tumor-implanted mice		(70)
*Salmonella typhimurium Dam(-), AroA(-)*	Breast cancer	Legumain	Suppressing tumor angiogenesis	BALB/c mice	D2F2	(91)
*Salmonella typhimurium* -lux	Hepatocellular and colon cancer	Mouse alpha-fetoprotein (AFP) gene	Promote protective immunity	BALB/c mice C57/J mice	CT26	(92)
*Salmonella typhimurium SL7207*	Prostate cancer	Prostate stem cell antigen (PCSA)	Generated specific antitumor immune responses	C57 BL/6 mice	TRAMPC1	(93)
*Salmonella typhimurium*	Lung carcinoma	DNA vaccine (pcDNA3.1-FLK1(ECD)	Prevented recurrence and metastasis	Lewis lung carcinoma model in mice		(94)
*Salmonella typhimurium SL7207*	Neuroblastoma	DNA vaccines	Improved cellular anti-NB immune response	Mice		(95)
*Salmonella typhimurium, MvP728 (purD/htrA)*	Colon carcinoma and orthotopic DBT glioblastoma	Survivin	Regulated T3SS of *Salmonella* and NKT ligands	Female BALB/c mice	CT26	(96)
*Salmonella SL3261*	Colorectal cancer	4-1BBL	Enhanced T cell immunity	Male Sprague Dawley (SD) rat model of colorectal tumor		(97)
*Salmonella*	Lung adenocarcinoma	RBM5	Apoptosis	BALB/c nude mice bearing A549 tumors	A549	(98)
*Salmonella typhimurium*	Mammary carcinoma	TRAIL	Reduced tumor growth	Mice	4T1	(99)
*Salmonella choleraesuis*	Melanoma and bladder tumor	Endostatin	Decreased intra tumoral microvessel density, reduced VEGF, CD8(+) T cell infiltration	BALB/c mice bearing 4T1 tumors		(100)
*Salmonella typhimurium*	Hepatocarcinoma	Stat3-siRNA and endostatin	Reduced cell proliferation, increased apoptosis, inhibited angiogenesis	HCC model in C57BL/6 mice		(101)
*Salmonella typhimurium*	Gastric cancer	Apoptin	Inhibited tumor growth	Mice		(102)
*Salmonella typhimurium*	Tumor	Diphtheria toxin A chain (DTA)	PLK1 reduction	Nude mice bearing human MDA-MB-231 xenografts		(103)
*Salmonella typhimurium*	B-cell lymphoma	CD40L	Prevented tumor growth	BALB/c mice		(104)
*Salmonella*	Melanoma	IL-18	Production of IFN gamma	Melanoma‐bearing mice		(105)
*Salmonella*	Melanoma	IL-4	Production of IFN gamma	Melanoma‐bearing mice		(105)
*Salmonella typhimurium SL3261*	Tumor	hGM-CSF	Increased cytotoxic T cells	BALB/c and C57BL/6 mice		(106)
*Salmonella typhimurium SL3261*	Tumor	hIL-12& mIL-12	Increased cytotoxic T cells	BALB/c and C57BL/6 mice		(106)
*Salmonella typhimurium*	Lymphoma	Herpes simplex virus thymidine kinase	Lymphoma reduced	Tumor-bearing C57/Bl6J-OlaHsd mice		(107)
*Salmonella*	Tumor	Cytosine deaminase	Inhibition of tumor growth			(74)
*Salmonella typhimurium TAPET-CD*	Tumor	Cytosine deaminase	Inhibition of tumor growth	Tumor-bearing mice		(108)
*Salmonella typhimurium*	Cervical cancer	SipB160/HPV16 E7	Secretion of INF-γ and TNF-α	Mice bearing TC-1 tumors	TC-1	(109)
*Salmonella typhimurium VNP20009*	Tumor	EGFR-targeted cytotoxic proteins	Induce apoptosis	Mice		(110)
*Salmonella typhimurium*	Colon or breast tumor cells	TGFα-PE38	Inhibition of solid tumor growth	Mice bearing colon or breast tumors		(57)
*Salmonella typhimurium*	Lymphoma cells	CD20	Tumor-specific response	Tumor-bearing C57/Bl6J-OlaHsd mice		(107)
*Salmonella typhimurium*	Human breast cancer and melanoma	RGD peptide	Targeting and therapeutic effects	Mice bearing human lymphomas	(MDA-MB-231)& (MDA-MB-435)	(75)
*Salmonella typhimurium VNP20009*	Tumors	Anti-CEA- scFv	Tumor accumulation of CD3(+) T cells and CD11b(+) macrophages	CEA transgenic mice	MC38CEA	(111)
*Salmonella typhimurium ST8*	Colon cancer	ST8/pSEndo	Necrosis and anti-angiogenesis	Tumor-bearing immunocompetent mice	CT26	(112)
*Salmonella typhimurium S634*	Colon carcinoma and melanoma	S636/pES	Apoptosis and anti-angiogenesis	Tumor-bearing mice	CT26 & B16F10	(113)
*Salmonella typhimurium*	Lymphoblastic leukemia	L-asparaginase		Tumor-bearing mice		(82)
*Salmonella typhimurium*	Colon cancer	FlaB	Activation of M1 macrophages and suppression ofM2	TLR4 and MyD88 knockout mice	(TLR5)-negative	(114)
*Salmonella typhimurium*	Breast and colon carcinoma	FasL	Reduced pulmonary metastases	BALB/c mice injected with D2F2 breast carcinoma cells	D2F2 or CT-26	(115)
*Salmonella typhimurium*	Mammary carcinoma	TRAIL	Apoptosis	Mice	4T1	(99)
*Salmonella typhimurium*	Colon and breast tumor	TGFα-PE38	Inhibition of tumor growth	Mice bearing colon or breast tumors		(57)
*Salmonella typhimurium*	Colon carcinoma	Noxa	Anti-cancer effect	Mice with CT26 colon carcinoma	CT26	(56)
*Salmonella typhimurium*	Tumor	Cytolysin A	Expression of reporter genes	BALB/c athymic nu−/nu− mice	(CT-26)	(55)
*Salmonella typhimurium*	Melanoma	IFN-γ	induced cytotoxicity	C57BL/6 mice bearing B16F10 melanoma	B16F10	(116)
*Salmonella typhimurium*	Multi-drug-resistant carcinomas	CCL21	Anti-tumor activity	Mice		(117)
*Salmonella typhimurium*	Tumors	LIGHT	Primary tumor inhibition and fewer pulmonary metastases	BALB/c mice bearing CT-26 colon carcinoma		(118)
*Salmonella typhimurium*	Subcutaneous tumors	IL-18	Primary tumor inhibition and fewer pulmonary metastases	BALB/c mice		(119)
*Salmonella typhimurium*	Melanoma	IL2	Decreased angiogenesis and increased necrosis	Mice	B16F1	(120)
*Salmonella typhimurium*	Osteosarcoma	IL-2	Reduced metastases	Female Balb/c mice	ATCC K7M2	(121)

## *Salmonella* Bacteria in Combination Therapies

Chemotherapy can block tumor cell division by interfering with microtubule assembly, disrupting cellular metabolism, or inhibiting DNA repair or replication (122). Several chemotherapy drugs, such as anthracyclines, also work by an immunologic mechanism by causing so-called “immunologic tumor cell death.” Moreover, chemotherapy may be able to inhibit immunosuppressive pathways occurring in the tumor thus releasing the immune attack to be more effective (123).

Saltzman et al. investigated the efficacy of bacterial therapy combined with chemotherapy against tumors (124) (**Table 2**). They used BALB-neu T mice with orthotopic mammary tumors resembling invasive human breast cancer driven by the Her2 oncogene. An attenuated strain of *S. typhimurium* was used in combination with doxorubicin at two doses, high (5 mg/kg) and low (1.25 mg/kg). *S. typhimurium* was injected intravenously on day 0 and doxorubicin days 0, 7, and 14. Mammary pad tumors were evaluated weekly up to day 35 to determine the efficacy, and mice were weighed for toxicity assessment. At day 35, the high dose of doxorubicin limited tumor growth to 1.4-fold, but the body weight had fallen by 25% due to severe toxicity. A single dose of *S. typhimurium* plus low dose doxorubicin (1.25 mg/kg) restricted the tumor growth to a somewhat lesser degree, but with only 5% weight loss showing no clinical toxicity (124).

**Table 2:** Various *Salmonella* strains that have been used in the combination therapy of cancer.

Salmonella species	Type of cancer	Combine with	Anti-cancer effect	Model	Type of cell line	Ref
*Salmonella typhimurium A1-R*	Human colon cancer	Bright-light surgery (BLS)	Increase survival	Athymic nu/nu nude mice	HT-29	(140)
*Salmonella typhimurium A1-R*	Mammary adenocarcinoma	Surgery	Inhibiting surgery-induced metastasis	Mice	4T1-RFP	(141)
*Salmonella typhimurium*	Melanoma	γ-radiation	Apoptosis	Tumor-bearing mice		(142)
*Salmonella typhimurium ΔppGpp*	Colon tumor	Radiotherapy	Reduced tumor growth	Colon tumor (CT26) model of BALB/c mice	CT26	(11)
*Salmonella ˗Lipid A*	Melanomas	X-rays	Produced supra-additive antitumor effects	Mice bearing B16F10 or Cloudman S91 melanomas	B16F10 or Cloudman S91	(143)
*Salmonella*	Mammary tumors	Lipid A	Robust intratumoral accumulation of Salmonella	Mice with 4T1 mammary tumors	4T1	(144)
*Salmonella typhimurium*	Breast cancer	Low dose chemotherapy	Decreases tumor burden and less toxic	BALB-neuT mice	Her2-driven	(124)
*Salmonella typhimurium LVR01*	Melanoma	Imiquimod	Enhancement the pro-inflammatory cytokines and chemokines	B16F1 melanoma-bearing mice	B16F1	(145)
*SalmonellatyphiSL7207*	Ovarian cancer	RNA interference	Slow tumor growth	SKOV-3/DDP tumor-bearing mice	SKOV-3/DDP	(45)
*Salmonella typhimurium A1-R*	Cancers	Cisplatinum (CDDP) or paclitaxel (PTX)	Prevented tumor growth	Nude mice		(146)
*Salmonella typhimurium A1-R*	Osteosarcoma	Recombinant methioninase (rMETase)	Inhibited tumor growth	Athymic nu/nu nude mice	PDOX	(126)
*Salmonella typhimurium A1-R*	Sarcoma	Doxorubicin (DOX)	Inhibited tumor growth	Athymic nu/nu nude mice	PDOX	(147)
*Salmonella typhimurium A1-R*	Melanoma	Temozolomide (TEM)	Inhibited tumor growth	Athymic nu/nu nude mice	PDOX	(148)
*Salmonella typhimurium A1-R*	Pancreatic cancer	Anti-vascular endothelial growth factor (VEGF) therapy	Reduced tumor weight	Male athymic (*nu/nu*) nude mice	MiaPaCa-2-GFP	(63)
*Salmonella typhimurium VNP20009*	Melanoma	Anti-angiogenesis therapy (triptolide	Necrosis and modulation of angiogenesis	Female C57BL/6 mice		(69)
*Salmonella typhimurium VNP20009*	Melanoma	(ABCB5)	Delay tumor growth	Mice	B16F10	(132)
*Salmonella typhimurium VNP20009*	Melanoma	Chemotherapy cyclophosphamide (CTX)	Decrease in tumor microvessel density and (VEGF) level	Murine melanoma model		(62)
*Salmonella typhimurium VNP20009*	Lung cancer	Sox2 shRNA	Inhibition of angiogenesis	B16F10 mice model	A549	(137)

Shi et al. designed a *S. typhimurium* strain for the transfer of plasmid vectors (103). This vector was designed to allow dual transcription of therapeutic molecules, which could either be cytotoxic proteins or short hairpin RNAs, within the nucleus or the cytoplasm of eukaryotic cells. The expression of the foreign gene in the bacteria was driven using the T7 RNA polymerase enzyme and dual promoters, and was continuously propagated using an autocatalytic feedback loop. They used attenuated *Salmonella* to express the gene coding for diphtheria toxin A chain within the hypoxic regions of the tumor, thereby eradicating the tumor in a mouse model. The results showed that 26% (n = 5/19) of the mice were cured and the remainder survived to the termination of the experiment. In a different experiment, nude mice with human MDA-MB-231 xenograft tumors were treated with another strain of *S. typhimurium* containing a shRNA-encoding plasmid that targeted a cell cycle-associated protein, polo-like kinase 1 (PLK1). Tests showed that, in tumors, the levels of PLK1 transcript were 62.5 ± 18.6% (p = 0.015) lower compared to the control group 3 weeks after injection of 5 × 10(6) CFU of ST4/pIKT-shPlk. They named this approach “inter-kingdom gene delivery” (103).

A study conducted by Nguyen et al. (2010) described a strain of attenuated *S. typhimurium* that was defective in guanosine 5’-diphosphate-3’-diphosphate, and engineered to express cytolysin A (a 34-kDa pore-forming hemolytic protein) as well as the bacterial luciferase gene lux to allow *in vivo* imaging (55). In order to avoid undesirable expression in the liver and spleen after tail vein injection, the PBAD promoter from the *E. coli* arabinose operon, which can be activated by the sugar l-arabinose was employed. Mice with CT-26 tumors could be successfully imaged, some tumors were eradicated, and metastases were reduced upon cytolysin gene induction (55).

Liu et al. combined the identical engineered *S. typhimurium* strain (ΔppGpp) with radiotherapy (RT) to treat CT26 tumors in BALB/c mice (11). Mice were subjected to RT treatment after *S. typhimurium* ΔppGpp/pBAD-ClyA injection, using a single dose of 7 Gy at days 1, 4, and 7 for a total of 21 Gy. The combination treatment reduced tumor growth compared with either treatment used alone, and furthermore the radiation was shown not to affect the actual bacteria themselves (11).

It has been shown that recombinant methioninase (rMETase) can act as an anti-tumor agent by targeting the altered methionine metabolism typical of cancer. rMETase could overcome gemcitabine-resistance and lead to regression in a patient-derived orthotopic xenograft (PDOX) nude mouse model of pancreatic cancer (125). The clinical survival rate in metastatic osteosarcoma is less than 20%. Researchers had previously designed a PDOX osteosarcoma mouse model using a lung metastasis from a patient with osteosarcoma who failed CDDP therapy (126). The tumor-targeting *S. typhimurium* strainA1-R is auxotrophic for the amino-acids leucine and arginine, and has been used to treat mouse xenografts of prostate, breast cancer, osteosarcoma, and lung metastases (127). AR-1 is able to induce chemoresistant cancer cells to move from the G0/G1 phase of the cell cycle into the S/G2/Mphase (128). Intravenously injected *S. typhimurium* AR-1 could be beneficially combined with orally administered rMETase, because rMETase can selectively act against cells in the S/G2 phase of the cell cycle. A study tested this approach in the PDOX model of CDDP-resistant metastatic osteosarcoma. Different groups of PDOX mice were exposed to the following treatments 14 days after inoculation: G1, control with no treatment; G2, CDDP (6 mg/kg, i.p. daily for 2 weeks); G3, rMETase (100 unit/mouse, i.p., daily for 2 weeks); G4, *S*. *typhimurium* A1-R (5 × 10(7) CFU/100 µl, i.v., weekly, for 2 weeks); G5, *S. typhimurium*A1-R combined with rMETase; G6, *S. typhimurium*A1-R combined with rMETase and CDDP. Except for CDDP alone, all the other treatments inhibited growth of tumor in comparison with G1 control. The triple combination was the most effective.

Yano et al. investigated whether S. *typhimurium* A1-R could decoy quiescent cancer cells in the cell cycle from G0/G1 to S/G2/M phase as demonstrated by fluorescent imaging mediated by a fluorescence ubiquitination cell cycle indicator (FUCCI) (125). They used sequential treatment of a model of FUCCI-expressing stomach cancer MKN45 *in vivo* in mice with *S. typhimurium* A1-R plus rMETase to selectively trap the decoyed cancer cells in S/G2 phase. This was followed by additional administration of cisplatin (CDDP) or paclitaxel (PTX) chemotherapy to kill the decoyed and trapped cancer cells. The combination led to the complete regression of tumors, thus demonstrating the effectiveness of this “decoy, trap and shoot” chemotherapy.

Melanoma is known as one of the most deadly types of skin cancer (129, 130). Although some chemotherapy drugs such as dacarbazine, cyclophosphamide, and temozolomide show some therapeutic benefit, other drugs such as nitrosoureas, taxanes, vinca alkaloids or platinum-related drugs have not shown any positive effects in randomized trials, and do not extend survival (131). Melanoma tumors contain cancer stem cells, with a long-lasting renewable capacity and expression of a multidrug resistance pump called ATP-binding cassette sub-family B member 5 (ABCB5). In one study by Zhang et al., the researchers treated melanoma-bearing mice with an attenuated *Salmonella* strain (VNP20009) that was engineered to express short hairpin RNA targeting the ABCB5 gene (132). They combined this treatment with the chemostherapy drug cyclophosphamide (CTX) which is a known substrate of ABCB5. Concomitant use of VNP20009-shABCB5 and CTX compared with VNP20009 alone, could synergistically delay tumor growth and extend survival in the B16F10 mouse model (132).

In one study, Chen et al. compared the effects of conventional VNP20009 monotherapy, highly attenuated Salmonella strain, and a combination therapy that used both triptolide and VNP20009 in a mouse melanoma model. They showed that triptolide improved the tumor colonization with VNP20009 through decreasing the numbers of infiltrated neutrophils, which led to a larger necrotic area in the melanoma. Furthermore, the combination therapy could suppress angiogenesis of the tumor by decreasing the VEGF expression in a synergistic manner, and retarding the growth of the melanoma (133).

Lung cancer is a leading cause of cancer death, and 85% of cases are non-small cell lung carcinomas (NSCLC) with an average 5-year survival of only 4% (134). SOX2 (sex determining region Y)-box 2 is a transcription factor that is essential for maintaining the pluripotency of undifferentiated embryonic stem cells and lung cancer stem cells (135). Regulating Sox2 suppresses the metastasis and growth of many cancer cells, so Sox2 is a potential target in cancer treatment (136).

In one study by Zhao et al., a *S. typhimurium* VNP20009 strain carrying a Sox2 shRNA was designed (137). They also used a polypeptide called HM-3, which is an 18-amino acid peptide generated by the fusion of the Arg-Gly-Asp (RGD) sequence to the C-terminus of an endostatin-derived peptide. The RGD domain in HM3 specifically recognized the αvβ3 integrin, which is over-expressed in tumor vasculature. The combination of HM-3 and *S. typhimurium* VNP20009 carrying shRNA targeting Sox2 was tested *in vitro* and *in vivo*. Cell invasion and colony formation assays using A549 human lung cancer cells showed that shRNA against Sox2 could reduce migration, and trigger apoptosis by increasing Bax expression, cleaving caspase 3, and decreasing Bcl2. A mouse xenograft model of A549 was used to show that the combination therapy of HM-3 and VNP20009 Sox2 shRNA, could inhibit angiogenesis and slow lung cancer growth (137).

Gao et al. developed an oxygen tolerant attenuated Salmonella strain (KST0650) using radiation mutation technology. Results showed that the oxytolerant KST0650 strain possessed 20-times higher replication activity in CT26 cancer cells and was less virulent than wild type Salmonella. Furthermore, KST0650 could migrate effectively into tumor tissues in mice. KST0650 was further equipped with a plasmid harboring a spliced form of the intracellular pro-apoptotic protein sATF6, and the expression of sATF6 was controlled by the radiation-inducible recN promoter. The new strain was named KST0652, in which sATF6 protein expression was induced in response to radiation in a dose-dependent manner. This strain was effectively delivered to cancer cells and tumor tissues *via* the Salmonella type III secretion system (T3SS). In addition, combination treatment with KST0652 plus radiation showed a synergistic anti-tumor effect in the murine tumor model with complete inhibition of tumor growth and protected against mouse death (138).

Miyake et al. developed the tumor-targeting bacteria *S. typhimurium* A1-R. In this study cervical-cancer tumor fragments were implanted orthotopically into the uterine cervix of nude mice. They showed that nab-paclitaxal combined with *S. typhimurium* A1-R significantly suppressed tumor growth compared to the untreated control group, while the nab-PTX alone and *S. typhimurium* A1-R alone groups did not show significant efficacy as monotherapy compared to the control group (139).

## Conclusions

The pathophysiology of solid tumors imposes severe barriers that prevent the penetration and accumulation of anti-tumor chemotherapy drugs. The application of therapeutic bacteria to treat cancer has a long history, but may only recently have been taken seriously. A variety of oncolytic bacteria have been described including *Mycobacterium bovis* (BCG), *Streptococcus* and *Serratia* species, *Listeria monocytogenes*, as well as *Salmonella* species as discussed in the present review. *Salmonella* species have been often studied because of the availability of tumor-selective strains that have been significantly attenuated in their virulence, so that they do not pose a major threat of systemic infection or collateral damage. Moreover the bacteria can be controlled after their administration, by using compounds that only affect the bacterial cells and not the host cells. According to many studies, *Salmonella*-mediated antitumor therapy has significantly helped to suppress tumors and achieve higher rates of survival in animal models. The benefits of this therapy include automatic self-targeting, as well as the possibility of genetic manipulation to produce newly engineered attenuated strains. Nevertheless, *Salmonella-*mediated anticancer therapy has not yet been clinically established, and is still faced with numerous practical challenges. For instance, somewhat unsatisfactory results were obtained in the phase I clinical trials that have so far been conducted. The changes in the tumor microenvironment produced by *Salmonella* are still not fully understood. The complicated interactions between Salmonella, inflammatory mediators, as well as the effects on host immunity are difficult to figure out Combinations of *Salmonella*-mediated approaches together with other types of tumor treatment could show synergistic benefits. BMCT alone will probably not be a complete substitute for traditional cancer therapy, but it may take part in new combination approaches to improve outcomes.

## Author Contributions

HM and MRH contributed in conception, design, statistical analysis and drafting of the manuscript. FB, MaS, KM, VT, MG, SAT, AK, MoH, MM-T, and AJ contributed in data collection and manuscript drafting. All authors contributed to the article and approved the submitted version.

## Conflict of Interest

MH declares the following potential conflicts of interest. Scientific Advisory Boards: Transdermal Cap Inc, Cleveland, OH; BeWell Global Inc, Wan Chai, Hong Kong; Hologenix Inc. Santa Monica, CA; LumiTheraInc, Poulsbo, WA; Vielight, Toronto, Canada; Bright Photomedicine, Sao Paulo, Brazil; Quantum Dynamics LLC, Cambridge, MA; Global Photon Inc, Bee Cave, TX; Medical Coherence, Boston MA; NeuroThera, Newark DE; JOOVV Inc, Minneapolis-St. Paul MN; AIRx Medical, Pleasanton CA; FIR Industries, Inc. Ramsey, NJ; UVLRx Therapeutics, Oldsmar, FL; Ultralux UV Inc, Lansing MI; Illumiheal&Petthera, Shoreline, WA; MB Lasertherapy, Houston, TX; ARRC LED, San Clemente, CA; Varuna Biomedical Corp. Incline Village, NV; Niraxx Light Therapeutics, Inc, Boston, MA. Consulting; Lexington Int, Boca Raton, FL; USHIO Corp, Japan; Merck KGaA, Darmstadt, Germany; Philips Electronics Nederland B.V. Eindhoven, Netherlands; Johnson & Johnson Inc, Philadelphia, PA; Sanofi-Aventis Deutschland GmbH, Frankfurt am Main, Germany. Stockholdings: Global Photon Inc, Bee Cave, TX; Mitonix, Newark, DE.

The remaining authors declare that the research was conducted in the absence of any commercial or financial relationships that could be construed as a potential conflict of interest.

## Abbreviation

5-FC, 5-Fluorocytosine; 5-FdUMP, 5-fluorodeoxyuridine monophosphate; 5-FdUTP, 5-fluorodeoxyuridine triphosphate; 5-FU, 5-fluorouracil; 5-FUTP, 5-fluorouridine triphosphate; ABCB5, ATP-binding cassette sub-family B member 5; AFP, fetoprotein; Bax, Bcl-2-associated X protein; BLS, bright-light surgery; BMCT, Bacterial-mediated cancer therapy; CCL21, Chr. Chemokine (C-C motif) ligand 21; CD31, cluster of differentiation 31; CDDP, cisplatinum; CEA, carcinoembryonic antigen; CTX, cyclophosphamide; DAMP, danger-associated molecular patterns; DOX, Doxorubicin; DTA, Diphtheria toxin A chain; EGFR, Epidermal growth factor receptor; eIF2B, Eukaryotic translation initiation factor 2B;FasL, fas ligand; FLK1, Fetal Liver Kinase 1; FUCCI, fluorescence ubiquitination cell cycle indicator; GCN2, general control nonderepressible 2; Her2, Human epidermal growth factor receptor 2; hGM-CSF, Human Granulocyte Macrophage Colony Stimulating Factor; hIL-12, human IL-12; HPV, human papilloma virus; IL, interleukin; INF-γ, interferon gamma; L-ASNase, asparaginase; MDR, multidrug resistance; MDR1, multidrug-resistance gene; MHC, Major histocompatibility complex; NSCLC, non-small cell lung carcinomas; OmpA, outer membrane protein A; P-gp, permeability glycoprotein; PAMP, pathogen-related molecular patterns; PCR, polymerase chain reaction; PCSA, Prostate stem cell antigen; PDOX, pancreatic cancer patient-derived orthotopic xenografts; PLK1, polo like kinase 1; PTX, paclitaxel; RBM5, RNA‐binding motif protein 5; RGD, Arg-Gly-Asp; RGD, Arginylglycylaspartic acid; rMETase, recombinant methioninase; RT, radiotherapy; scFv, single-chain variable fragment; shRNA, short hairpin RNA; siRNA, small interfering RNA; Sox2, sex determining region Y; Stat3, Signal transducer and activator of transcription 3; TAM, tumor-associated macrophages; TEM, Temozolomide; TGFα, Transforming growth factor alpha; TNFα, Tumor necrosis factor; TRAIL, TNF-related apoptosis-inducing ligand; tRNA, transfer ribonucleic acid; VEGF, Vascular endothelial growth factor; αvβ3, alpha-v beta-3.
